# The identification of viral ribonucleotide reductase encoded by ORF23 and ORF141 genes and effect on CyHV-2 replication

**DOI:** 10.3389/fmicb.2023.1154840

**Published:** 2023-04-18

**Authors:** Wenjie Cheng, Qikang Chen, Yilin Ren, Ye Zhang, Liqun Lu, Lang Gui, Dan Xu

**Affiliations:** ^1^College of Fisheries and Life Science, Shanghai Ocean University, Shanghai, China; ^2^National Pathogen Collection Center for Aquatic Animals, Shanghai Ocean University, Shanghai, China; ^3^Key Laboratory of Freshwater Aquatic Genetic Resources, Ministry of Agriculture and Rural Affairs, Shanghai Ocean University, Shanghai, China; ^4^National Demonstration Center for Experimental Fisheries Science Education, Shanghai Ocean University, Shanghai, China

**Keywords:** CyHV-2, ORF23, ORF141, ribonucleotide reductase, hydroxyurea

## Abstract

**Introduction:**

Ribonucleotide reductase (RR) is essential for the replication of the double-stranded DNA virus CyHV-2 due to its ability to catalyze the conversion of ribonucleotides to deoxyribonucleotides, and is a potential target for the development of antiviral drugs to control CyHV-2 infection.

**Methods:**

Bioinformatic analysis was conducted to identify potential homologues of RR in CyHV-2. The transcription and translation levels of ORF23 and ORF141, which showed high homology to RR, were measured during CyHV-2 replication in GICF. Co-localization experiments and immunoprecipitation were performed to investigate the interaction between ORF23 and ORF141. siRNA interference experiments were conducted to evaluate the effect of silencing ORF23 and ORF141 on CyHV-2 replication. The inhibitory effect of hydroxyurea, a nucleotide reductase inhibitor, on CyHV-2 replication in GICF cells and RR enzymatic activity *in vitro* was also evaluated.

**Results:**

ORF23 and ORF141 were identified as potential viral ribonucleotide reductase homologues in CyHV-2, and their transcription and translation levels increased with CyHV-2 replication. Co-localization experiments and immunoprecipitation suggested an interaction between the two proteins. Simultaneous silencing of ORF23 and ORF141 effectively inhibited the replication of CyHV-2. Additionally, hydroxyurea inhibited the replication of CyHV-2 in GICF cells and the *in vitro* enzymatic activity of RR.

**Conclusion:**

These results suggest that the CyHV-2 proteins ORF23 and ORF141 function as viral ribonucleotide reductase and their function makes an effect to CyHV-2 replication. Targeting ribonucleotide reductase could be a crucial strategy for developing new antiviral drugs against CyHV-2 and other herpesviruses.

## 1. Introduction

More than 14 herpesviruses have been known to be associated with disease outbreaks in fish (Moshe et al., 2011), among which Cyprinid herpesvirus 2 (CyHV-2), a member of Alloherpesviridae family belonging to the genus *Cyprinivirus*, is a highly pathogenic double-stranded DNA virus causing necrosis of hematopoietic organs in goldfish (*Carassius auratus*) and crucian carp (*Carassius auratus gibelio*). Since it was first discovered in Japan in 1992, the virus has spread rapidly around the world, including the United States (Goodwin et al., 2006), the United Kingdom (Ito et al., 2013), India (Sahoo et al., 2016), and so on. In 2012, CyHV-2 was also detected in carp farms in Yancheng, Jiangsu province (Xu et al., 2013), and the viral disease has caused serious damage to the global crucian carp industry.

Due to its large genome size (290 kbp), the DNA synthesis is important for CyHV-2 replication. The ribonucleotide reductase (RR) which consists of two distinct homodimers, large (RR1) and small (RR2) subunits is the only rate-limiting enzyme for DNA synthesis and plays an important role in viral replication *in vivo* and *in vitro* (Brandt et al., 1991; Boldogkói et al., 1996; Crump, 2018). The interaction between the subunits RR1 and RR2 is the premise of the activity of this enzyme (Aye et al., 2015).

Genome-wide prediction and analysis of proteins has reported in three types of Cyprinid herpesviruses (Davison et al., 2013), among which CyHV-2 contains about 150 open reading frames (ORFs) encoding functional proteins. However, the function of these predictive proteins remains unclear and their potential impact on CyHV-2 replication has not been clarified. ORF23 and ORF141 proteins of CyHV-2 (Tang et al., 2020) were found to have high homology with small subunits and large subunits of RR from other herpesviruses, and ORF141 also includes the nucleotide binding site, which predicted that they may function as two subunits of RR, respectively. Identification and functional analysis of ORF23 and ORF141 proteins of CyHV-2 helps to further understand their potential effect on CyHV-2 replication.

Selective inhibition of RR was reported to be an effective anti-herpesvirus strategy (Sukhada et al., 2013; Dai et al., 2017). Hydroxyurea, a small subunit inhibitor, has been widely used in clinical cancer therapy by inhibiting the hRR2 tyrosine group and the catalytic effect of the total hRR complex (Lassmann et al., 1992; Nigović et al., 2005).

Currently, there is no information available regarding the roles of the CyHV-2 proteins ORF23 and ORF141 and the potential use of hydroxyurea in the treatment of viral diseases of fish. In order to better understand the molecular biological function and pathophysiology of CyHV-2, this study first characterized the properties and functions of ORF23 and ORF141 proteins of CyHV-2, and also provided novel and significant data for further clarification of the mechanism of CyHV-2 infection. At the same time, a preliminary study of hydroxyurea in the treatment of CyHV-2 infection was carried out to lay a foundation for the development of effective therapeutic drugs.

## 2. Materials and methods

### 2.1. Cells culture and viral infection

*Carassius auratus gibelio* caudal fin (GICF) cell line was established in our laboratory (Lu et al., 2018). GICF cells were cultured in M199 medium (Gibco, USA) supplemented with 10% fetal bovine serum (Gibco, USA) and antibiotics (100 U penicillin ml^–1^ and 100 mg streptomycin ml^–1^) at 27°C. HEK293T cells were cultured in DMEM Medium (Gibco, USA) supplemented with 10% fetal bovine serum (Gibco, USA) at 37°C and 5% CO_2_. The CyHV-2 (YC01) strain was isolated from infected *C. auratus gibelio* in Sheyang, Jiangsu province, China (Xu et al., 2013). Transfer GICF cells to a 6-well plate and seal the plate. When the cells are confluent in the wells, remove the original medium and add CyHV-2 virus diluted with M199 medium (MOI = 1). Incubate at 25°C for 1 h, then remove the virus solution and add M199 medium with 2.5% FBS. Continue to culture at 25°C and observe and record the cell morphology every 24 h.

### 2.2. Construction of different recombinant plasmids with CyHV-2 ORF23 and ORF141

Cyprinid herpesvirus 2 ORF23 and ORF141 genes were amplified from viral genome by using primers shown in Supplementary Table 2. The recombinant plasmids pDsRed-N1-ORF23, pCMV-ORF23, pEGFP-N1-ORF141, and pcDNA3.1-ORF141 were constructed by cloning restriction endonuclease digested PCR products into vectors pDsRed-N1, pCMV-HA, pEGFP-N1, and pcDNA3.1-3 × FLAG at the indicated sites, respectively. pDsRed-N1-ORF23 and pEGFP-N1-ORF141 were used for analysis of the subcellular localization of ORF23 and ORF141, and pCMV-ORF23 and pcDNA3.1-ORF141 for analysis of interactions between ORF23 and ORF141. All plasmids used in this study were verified by sequencing.

### 2.3. RNA and DNA preparation and real-time quantitative PCRs

Total RNA was extracted from GICF cells using TRIzol kit (Takara, Japan), and then cDNA was generated using cDNA synthesis kit (Takara, Japan) according to the manufacturer’s instructions. mRNAs of ORF23 and ORF141 genes were quantified by real-time polymerase chain reaction (RT-qPCR) using SYBR green chemistry (Takara, Japan) and normalized to β-*actin* mRNA levels. The detection of CyHV-2 copies was performed as described previously (Xu et al., 2014). Total viral DNA was extracted using a Tissue Genomic DNA Isolation Kit (Tiangen, China). The RT-qPCR conditions were as follows: 95°C for 30 s, followed by 39 cycles at 95°C for 5 s, 60°C for 30 s. Sequences of primers used for RT-qPCR are described in Supplementary Table 2. Real-time assays were performed in a CFX96™ Real-Time PCR Detection System (Bio-Rad, USA).

### 2.4. Western blot analysis

*Carassius auratus gibelio* caudal fin cells were collected and lysed in RIPA lysis buffer (50 mM Tris, 150 mM NaCl, 1% Triton X-100, 1% sodium deoxycholate, 0.1% SDS, pH 7.4; Beyotime, China). The extracted cellular proteins were separated in 10% SDS-PAGE gels using a Mini-PROTEAN^®^ 3 Cell (Bio-Rid) system, and then transferred onto a polyvinylidene difluoride (PVDF) membrane using the Trans-Blot^®^ SD Semi-Dry Transfer Cell system (Bio-Rad, USA). The membranes were subsequently blocked with phosphate buffered solution (PBS) containing 5% (w/v) non-fat milk at room temperature (RT) for 2 h and then incubated overnight at 4°C with primary antibodies (1:1,000 dilution). Primary antibodies include: mouse polyclonal antibodies ORF23 and ORF141 were prepared by our laboratory (antibody potency analyses can be seen in Supplementary Figures 1, 2, and information on pertinent recombinant plasmids and primers can be found in Supplementary Table 2). After washed with PBST, the cells were incubated with HRP-labeled Goat Anti-Mouse IgG as the secondary antibody (Abbkine, China, 1:5,000 dilution) for 2 h at RT, and then washed with PBST. The proteins were visualized by chemiluminescent method using the ECL and detected using ChemiDoc™ Imaging System (Bio-Rad, USA).

### 2.5. Indirect immunofluorescence assay

The GICF cells grown to approximately 95% confluence in 6-well microplates were infected with CyHV-2 (MOI = 1), and uninfected GICF cells served as a negative control. At 96 h post-transfection (hpt), the medium was aspirated and the cells were washed with PBS. After fixed with 4% paraformaldehyde for 15 min at RT, cells were blocked with 3% bovine serum albumin (BSA) overnight at RT. After aspiration of the BSA solution, the cells were incubated with the prepared polyclonal antibody as primary antibody (1:200 dilution) for 2 h at RT. After washed with PBST, the cells were incubated with DyLight 488 and 649, Goat Anti-Mouse IgG as the secondary antibody (Abbkine, China, 1:200 dilution) for 2 h at RT, and then washed with PBST. DAPI stain was added and incubated for 15 min at RT, and then PBS was added for washing. After the addition of anti-fluorescence attenuator, fluorescence was measured using a fluorescent inverted microscope (IX71, Olympus, Japan).

### 2.6. Co-localization assay

The HEK293T cells grown to approximately 80% confluence in 6-well microplates were transiently transfected with indicated plasmids (1.5 μg per plasmid). At 48 hpt, the cells were rinsed with PBS, and then fixed in 4% paraformaldehyde for 15 min at 4°C. Subsequently, the cells were rinsed with PBS, and the cellular nuclei were stained with DAPI for 15 min. After rinsing and adding anti-fluorescence attenuation blocker, the stained cells were observed by confocal fluorescence microscopy (SP8, Leica, GER).

### 2.7. Ribonucleotide reductase activity assay

Ribonucleotide reductase assay was performed in 50 mM HEPES-KOH buffer (5 mM magnesium chloride, 50 mM dithiothreitol, and 20 mM ferrous ammonium sulfate) containing effectors and substrates as follows: 2.7 mM ATP, 175 μM dATP, 80 μM dTTP, 125 μM dGTP, 80 μM CDP, 90 μM UDP, 250 μM ADP, and 130 μM GDP. After 1 h incubation at 4°C, 100 μl of the above reaction solution was removed and the reaction was stopped by adding 50% perchloric acid. Immediately after the addition of perchloric acid, the samples were vortexed and placed on ice. After 5 min on ice, KOH was added to adjust the pH to 8–9. After centrifugation, the recovered supernatant was subjected to chromatography on a borate resin. One milliliter of borate resin was equilibrated with a buffer containing 15 mM magnesium chloride and 0.15 M ammonium bicarbonate and packed into the column. A total of 200 μl of the recovered supernatant supplemented with 2 M Mgcl_2_ was loaded onto the boric acid column and washed with 1.5 ml of buffer. The eluate was collected for HPLC analysis. dCDP in the eluate was separated using a HPLC equipped with a PartiSphere-10 SAX column and detected at 260 nm UV at a flow rate of 1.5 ml/min in a 0.15 M ammonium phosphate mobile phase.

### 2.8. Co-immunoprecipitation assay

HEK293T cells grown to approximately 80% confluency in 6-well plates were co-transfected with indicated plasmids (1.5 μg per plasmid). At 24 hpt, the cells were lysed with RIPA lysis buffer (Beyotime, China) containing 1 mM PMSF for 1 h on ice. The cell lysates were centrifuged at 10,000 × *g* for 15 min at 4°C, and the collected supernatants were used for co-immunoprecipitation (co-IP) assays. The supernatants were incubated with anti-HA beads pre-washed with lysis buffer under continuous shaking at 4°C for 1 h. After the beads were rinsed with PBS (pH = 7.4) three times (20 min/time) under continuous shaking at 4°C, the immunoprecipitated proteins were released by boiling in water and then subjected to a Western blot analysis.

### 2.9. Cell viability and hydroxyurea inhibition assays

*Carassius auratus gibelio* caudal fin cells were transferred to a 6-well cell culture plate, and 0, 10, 20, 40, 60, 80, and 100 μM of hydroxyurea (127-07-1, MCE, USA) solutions prepared in 10% M199 medium were added after cells were spread over 90% of the plate. After 5 days of incubation at 25°C in a constant-temperature incubator (SANYO, Japan), the cells were collected by trypsin digestion, mixed with the Muse Count & Viability Kit (Merck, USA), incubated at RT in the dark for 5 min, and then the Guava^®^ Muse^®^ Cell Analyzer (Merck, USA) was used to analyze the viability of the cells. For *in vitro* inhibition assays, CyHV-2-infected GICF cells (MOI = 1) were treated with 10, 20, 40, 60, and 80 μM of hydroxyurea and the effect of hydroxyurea on viral replication was analyzed by measuring expression of the nucleocapsid protein (ORF72) at mRNA and protein levels.

### 2.10. Statistical analysis

All data were analyzed by one-way ANOVA to calculate the means and standard error of mean (SEM) for triplicate assays, and the data were conducted using Student’s *t*-test, and *P* < 0.05 or <0.01 was considered statistically significant. All statistical analysis was performed with SPSS 16.0 (SPSS, USA).

## 3. Results

### 3.1. Phylogenetic tree analysis and protein domain prediction of ORF23 and ORF141

**FIGURE 1:**
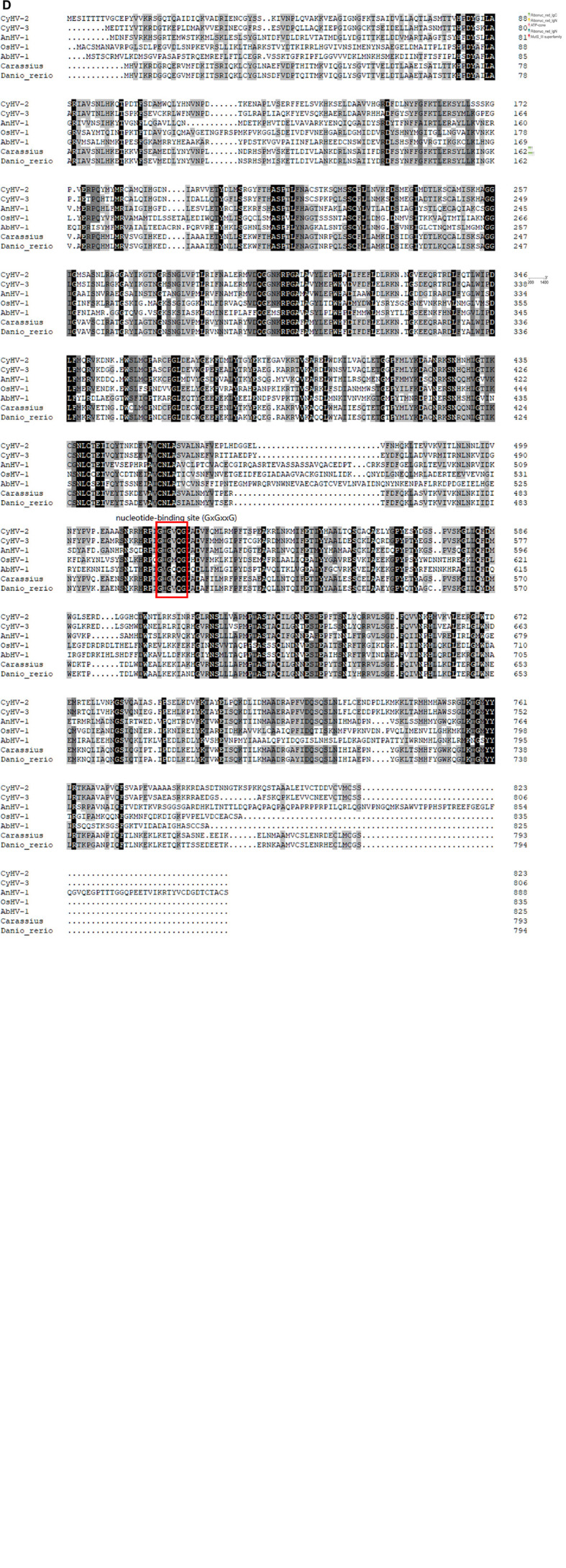
Phylogenetic tree analysis and protein domain prediction of ORF23 and ORF141. Amino acid sequences were obtained from Uniprot website (https://www.Uniprot.org/). **(A,B)** The phylogenetic tree was constructed based on the amino acid sequences of CyHV-2 ORF23 and ORF141, by using the neighbor-joining method in MEGA11.0 software. The evolutionary tree differentiation node shows 1,000 bootstrap replications. Smart (http://SMART.embl-heidelberg.de/) software was used to analyze the protein structure, and different protein domain were labeled with regions in different colors. **(C)** Alignment of CyHV-2 ORF23 with full-length among other species and herpesviruses, by using DNAman 8.0 software. **(D)** Alignment of CyHV-2 ORF141 with full-length among other species and herpesviruses, nucleotide binding site was marked in red box-labeled.

A phylogenetic tree was constructed based on the amino acid sequences of CyHV-2 ORF23 and ORF141 (the results of the statistical analysis related to the phylogenetic tree are presented in Supplementary Figures 3, 4), and protein structure domain prediction was performed. The results showed that CyHV-2 ORF23 and ORF141 shared high homology with the RRs from two species of fish (*C. auratus* and *Danio rerio*) and those from Abalone herpesvirus (AbHV) and Anguilla herpesvirus (AngHV) belonging to the family Alloherpesviridae (Figures 1A, B), indicating that CyHV-2 ORF23 and ORF141 were closely related to the RRs of fish and the family Herpesviridae. Figures 1C, D show protein homology analysis, where CyHV-2 ORF141 was predicted to possess the same nucleotide binding site as other RRs, suggesting that the proteins encoded by ORF23 and ORF141 could function as RR in CyHV-2.

### 3.2. Indirect immunofluorescence assay of CyHV-2 ORF23 and ORF141

**FIGURE 2:**
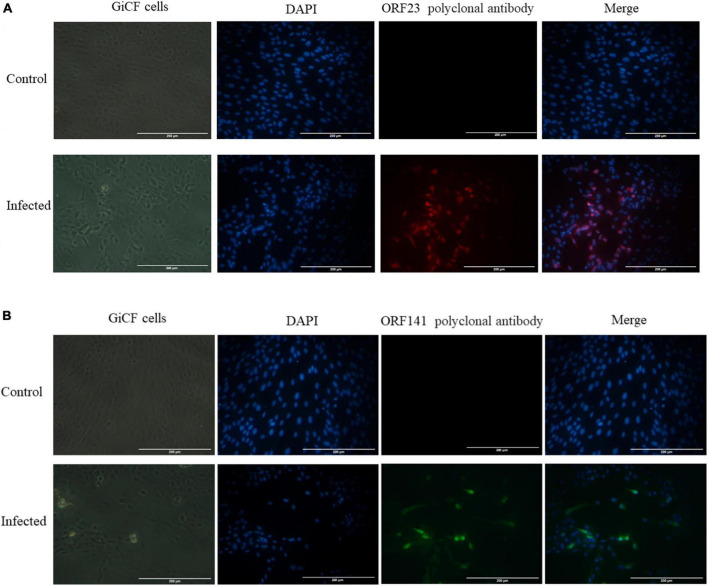
Indirect immunofluorescence detection of ORF23 and ORF141 in GICF cells infected with CyHV-2 (MOI = 1). **(A,B)** The control group was uninfected GICF cells, while the infected group was the GICF cells infected with CyHV-2 (MOI = 1). The nuclei were stained with DAPI. **(A)** ORF23 pAb was labeled in red by secondary antibody coupled with red fluorescent substrates; **(B)** ORF141 pAb was labeled in green by secondary antibody coupled with green fluorescent substrates. Scale bars indicate 200 μm **(A,B)**.

Uninfected and CyHV-2-infected (MOI = 1) GICF cells were fixed and permeabilized with 4% paraformaldehyde. The cells were incubated with mouse anti-CyHV-2 ORF23 and ORF141 polyclonal antibodies as primary antibodies and HRP-labeled Goat Anti-Mouse IgG as secondary antibodies. HRP-labeled secondary antibody coupled with red/green fluorescent substrate, with red used to label ORF23 and green used to label ORF141. Specific red fluorescence signal and green fluorescence signal were observed in CyHV-2-infected GICF cells, but not in uninfected GICF cells (Figures 2A, B), which suggested that the prepared polyclonal antibodies specifically recognize CyHV-2 virus and ORF23 and ORF141 proteins were distributed in the nucleus and cytoplasm of GICF cells during CyHV-2 infection.

### 3.3. Expression of ORF23 and ORF141 during CyHV-2 virus infection

**FIGURE 3:**
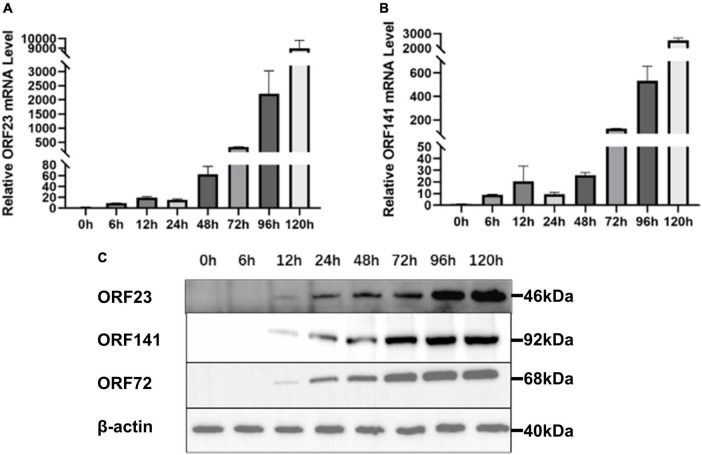
Expression mRNA and translation levels of ORF23 and ORF141 in GICF during CyHV-2 infection. **(A)** Total RNA of GICF cells infected by CyHV-2 (MOI = 1) was extracted at 0, 6, 12, 24, 48, 72, 96, and 120 hpi, the relative expression of *ORF23* was detected by RT-qPCR with β-*actin* as internal control. **(B)** Total RNA of GICF cells infected by CyHV-2 (MOI = 1) was extracted at 0, 6, 12, 24, 48, 72, 96, and 120 hpi, the relative expression of ORF141 was detected by RT-qPCR with β-*actin* as internal control. **(C)** ORF23, ORF141, and ORF72 protein expression was positively correlated. The total protein of GICF cells infected CyHV-2 (MOI = 1) was collected at 0, 6, 12, 24, 48, 72, 96, and 120 hpi and detected by Western blot.

Real-time polymerase chain reaction and Western blotting were used to detect the temporal expression patterns of ORF23 and ORF141 in GICF cells during infection of CyHV-2. CyHV-2 capsid protein ORF72 was stably expressed in host cells and used as a reference for viral copies in previous studies. As shown in Figures 3A, B mRNA of *ORF23* or *ORF141* was first detected in GICF cells at 6 hpi, but ORF23 or ORF141 protein was not detected in GICF cells until 12 hpi. Both mRNA and protein expression of ORF23 and ORF141 reached to their maximum levels in GICF cells at 120 hpi. As a control, ORF72 protein was first detected in GICF cells at 12 hpi, and its levels gradually increased thereafter (Figure 3C).

### 3.4. Location and interaction of CyHV-2 ORF23 and ORF141

**FIGURE 4:**
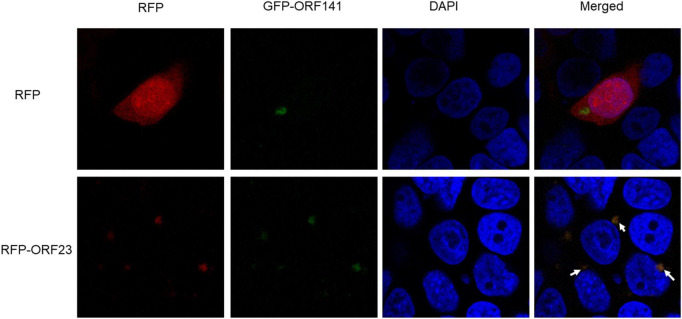
Subcellular localization of ORF23 and ORF141. The control group HEK293T cells was transfected with pDsRed-N1 and pEGFP-N1-ORF141. The experimental group HEK293T cells was transfected with pDsRed-ORF23 and pEGFP-ORF141. Arrows indicate colocalization.

In order to examine the intracellular co-localization of ORF23 and ORF141, HEK293T cells were cotransfected with plasmids pDsRed-N1 and pEGFP-N1-ORF141 in the control group, and cotransfected with plasmids pDsRed-N1-ORF23 and pEGFP-N1-ORF141 in the experimental group. At 48 hpi, the subcellular localization was analyzed under confocal fluorescence microscopy. In the control group, a perinuclear accumulation of pEGFP-N1-ORF141 (green) was observed, whereas pDsRed-N1 (red) was uniformly distributed throughout the cytoplasm as well as in the nucleus. The control group did not show orange fluorescent signal (yielded by the overlap of red and green ones), which indicated that pDsRed-N1 and pEGFP-N1-ORF141 did not interact with each other. Contrastly, pDsRed-N1-ORF23 and pEGFP-N1-ORF141 clearly clustered near the nucleus and showed orange fluorescent signal at the clusters in the experimental group (Figure 4, arrows), which demonstrated that pDsRed-N1-ORF23 and pEGFP-N1-ORF141 were highly overlapping and revealed potential interaction between ORF23 and ORF141 proteins (Figure 4).

**FIGURE 5:**
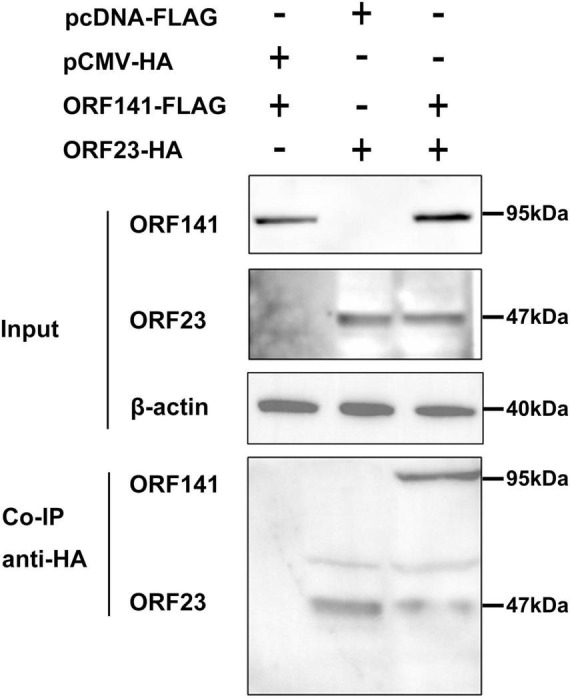
Determination of the interaction of ORF23-HA and ORF141-FLAG by co-immunoprecipitation. HEK293T cells were co-transfected with pCMV-ORF23 and pcDNA3.1-ORF141. After 48 h, the protein was extracted and co-immunoprecipitated using HA-tagged antibody. Subsequently, Western blot analysis was performed by elution. The β-*actin* was used as a loading control.

Meanwhile, the interaction between CyHV-2 ORF23 and ORF141 were examined by co-IP. HEK293T cells were co-transfected with plasmids with combinations shown in Figure 5, followed by co-IP. The immunoprecipitates from cells transiently expressing ORF23-HA and ORF141-FLAG were subjected to a Western blot analysis. The results indicated that ORF23-HA was specifically co-immunoprecipitated with ORF141-FLAG, suggesting an interaction between ORF23 and ORF141 (Figure 5).

### 3.5. CyHV-2 encoded ribonucleotide reductase activity assay

The peak areas of a series of dCDP standard solutions with different concentrations were measured and the standard curves were constructed by plotting the peak areas versus the concentration of dCDP standards (Supplementary Figure 5). The catalytic activity of RR was evaluated by the yields of dCDP which were calculated from the peak area using standard curve.

**FIGURE 6:**
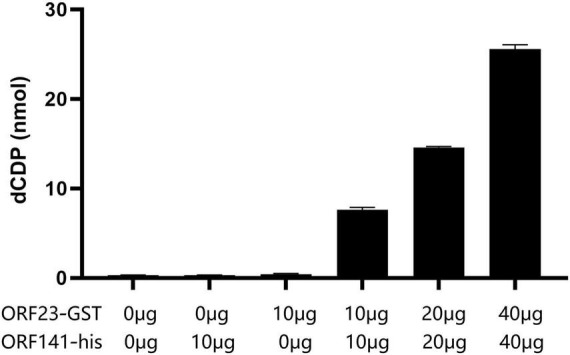
Ribonucleotide reductase activity assay. The amount of dCDP produced by adding different ORF23 and ORF141 components and different protein weight.

As shown in Figure 6, the dCDP levels were undetectable in the reaction solution when ORF23-GST or ORF141-His protein (relevant recombinant plasmids and primer information are provided in Supplementary Table 1) was added individually, but detectable in the reaction solution when ORF23-GST and ORF141-His were added simultaneously. The dCDP levels increased gradually in the reaction solution as the amount of added ORF23-GST and ORF141-His proteins increased. It was indicated that the interaction between ORF23-GST and ORF141-His proteins is essential for catalyzing the reduction of CDP to dCDP.

### 3.6. Inhibition of CyHV-2 replication by siRNA interference of ORF23 and ORF141

**FIGURE 7:**
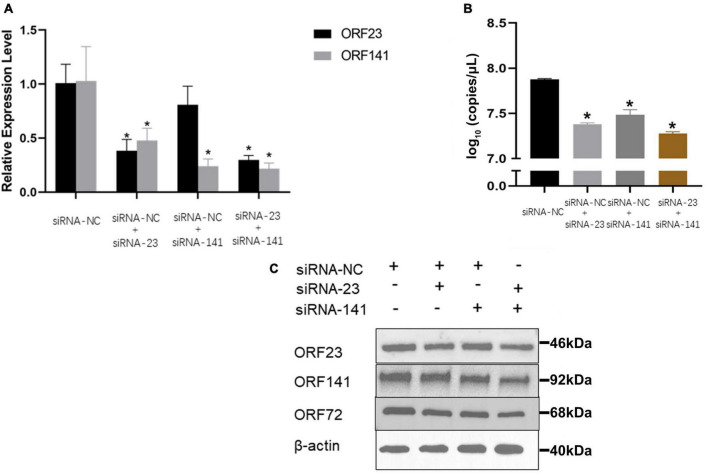
The effect of different siRNA transfection on the relative expression of ORF23 and ORF141 and viral replication. **(A)** The transcription levels of ORF23 and ORF141 mRNA were detected 72 hpi after infection. The β-*actin* as a reference gene. **(B)** Effect of transfection of different siRNAs on copies number of CyHV-2 in cell supernatant 96 hpi. **(C)** Changes of ORF72, ORF23, and ORF141 protein levels in transfected cells at 96 hpi. Protein normalization results in Supplementary Figure 6. The β-*actin* is used as a loading control. Mean ± SEM shows representative data sets from three independent experiments, *P < 0.05 vs. NC group.

The mRNA transcription and protein expression levels of ORF23 or/and ORF141 proteins were significantly decreased in infected GICF cells transfected with siRNA-ORF23 or/and siRNA-ORF141 (experimental groups) compared to GICF cells transfected with siRNA-NC (the control group) relevant sequences are shown in Supplementary Table 2. The results of qPCR and Western blot also showed that the viral copy number and protein expression of CyHV-2 in experimental groups were lower than those in the control group. These findings suggested that simultaneous silencing of ORF23 and ORF141 expression can significantly inhibit the replication of CyHV-2 (Figure 7).

### 3.7. The safe concentration of hydroxyurea on GICF cells

**FIGURE 8:**
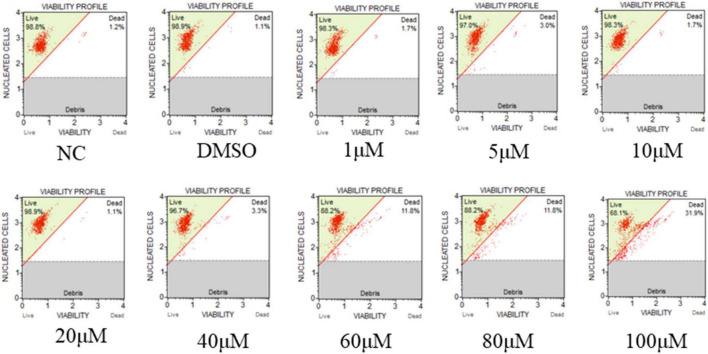
Effect of hydroxyurea on GICF cell. The effects of different dosages of hydroxyurea (0, 1, 5, 10, 20, 40, 60, 80, and 100 μM) on GICF cells were observed by microscope, and the activity of GICF cells was determined by Guava^®^ Muse^®^ cell analyzer.

Due to the importance of RR for viral replication, hydroxyurea, an inhibitor of RR, was selected to test its inhibitory effect on CyHV-2 and evaluated its potential antiviral effect in GICF cells. The viability of GICF cells exposed to graded doses of hydroxyurea was analyzed by using cell viability assay and microscopic observation. Microscopic observation (Figure 8; lower panel) showed that cell death was evident at the highest concentration of hydroxyurea (100 μM). GICF cells exposed to hydroxyurea with the dose range of 0–80 μM had viabilities of 88.2∼98.9%, which was higher than 85%. However, the survival rate of GICF cells treated with 100 μM of hydroxyurea was 68.1%, which was lower than 85% (Supplementary Figure 7). Therefore, the safe dose range of hydroxyurea for GICF cells was considered to be 0∼80 μM.

### 3.8. Effect of hydroxyurea on CyHV-2 replication and its encoded ribonucleotide reductase

**FIGURE 9:**
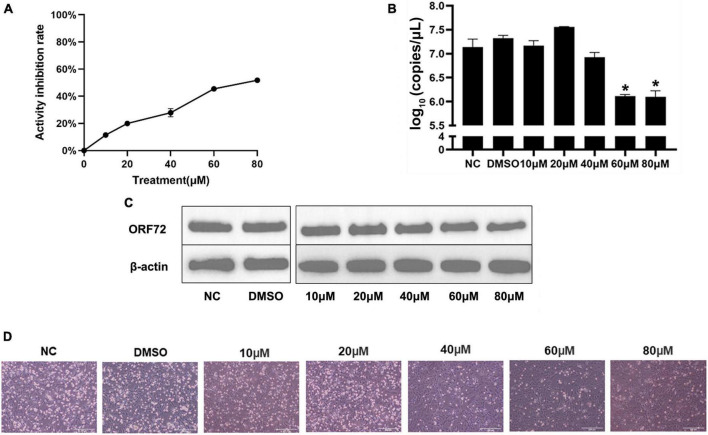
Hydroxyurea inhibited CyHV-2 replication and protein expression. **(A)** Inhibitory effect of hydroxyurea on ribonucleotide reductase, and the dosage of both recombinant proteins is 10 μg. **(B)** Changes of CyHV-2 copy number in virus infected (MOI = 1) cells treated with 0, 10, 20, 40, 60, and 80 μM hydroxyurea. **(C)** The nucleocapsid protein expression of CyHV-2 ORF72 under 0, 10, 20, 40, 60, and 80 μM hydroxyurea treatment. **(D)** Status of CyHV-2 infected (MOI = 1) cells under 0, 10, 20, 40, 60, and 80 μM hydroxyurea treatment. Mean ± SEM shows representative data sets from three independent experiments, *P < 0.05 vs. NC group.

As showed in the Figure 9A, hydroxyurea inhibited the activity of CyHV-2 RR, and the inhibition was enhanced with the increase of hydroxyurea concentration. Activity of CyHV-2 RR reduced by 52% when the concentration of hydroxyurea reached to 80 μM.

Suppression of viral replication was analyzed at the transcriptional and translational levels using CyHV-2-infected (MOI = 1) GICF cells treated with hydroxyurea at the concentrations of 0, 10, 20, 40, 60, and 80 μM. RT-qPCR assay results (Figure 9B) showed that the mRNA levels of ORF72 gene was significantly reduced in CyHV-2 infected GICF cells treated with hydroxyurea at the concentration of 60 and 80 μM compared with those in untreated or DMSO-treated GICF cells, which was further confirmed in protein levels by Western blot assays (Figure 9C). Figure 9D shows the changes in the status of cells infected with virus after different concentrations of hydroxyurea treatment of GICF. It can be observed that in the safe concentration range, cell death decreases as hydroxyurea concentration increases. In summary, these results suggested that hydroxyurea could effectively inhibit the replication of CyHV-2 in GICF cells at concentrations of 60–80 μM.

## 4. Discussion and conclusion

Large DNA viruses require dNTPs as substrates for viral genome replication in host cells. However the concentrations of dNTPs are relatively low in the G0 and G1 cell cycle phases (Mathews, 2015). Large DNA viruses need to evolve strategies to increase the availability of dNTPs, such as HSV-1 which encodes a RR that catalyzes the conversion of ribonucleotides (NTPs) to deoxyribonucleotides (dNTPs) in host cells (Goldstein and Weller, 1988). The reduction of NTPs largely depends on the activity of RR which catalyzes the reduction by proton-coupled electron transfer reactions (Offenbacher and Barry, 2020). The reaction catalyzed by RR is an irreversible and critical step in the process of establishing a free nucleotide pool. RRs can be divided into class I, class II, and class III. The class I RRs, mainly found in prokaryotes and eukaryotes, have been extensively studied in *Escherichia coli*, *Saccharomyces*, *Mus musculus*, and *Homo sapiens* (Conner et al., 1994). Class I RRs consist of two homodimeric subunits that perform catalytic functions cooperatively. The small subunit of RR, a homodimer called β_2_, contains a di-iron cofactor, and tyrosyl radical. The large subunit of RR, also known as the α_2_ homodimer, contains an active catalytic site and two allosteric regulatory sites. For example, the active form of RR found in *E. coli* is the α_2_β_2_ dimer (Minnihan et al., 2013). This configuration is the basis of RR activity in almost all organisms. The free radicals are transferred from the β_2_ subunit to the α_2_ subunit and generate thiol radicals on the α_2_ subunit and catalyze the reduction of NDP to dNDP (Nordlund and Reichard, 2006).

Phylogenetic analysis and conserved region prediction of amino acid sequence indicated that ORF23 and ORF141 proteins of CyHV-2 are the homologs of small and large subunit of RR respectively and ORF141 have a nucleotide binding site, which suggested that both ORF23 and ORF141 proteins might function as RR in CyHV-2. In this study, ORF23 and ORF141 proteins of CyHV-2 were found to coexist in the region near the nucleus which can prove that they are spatially closer and most likely interacted with each other. And the interaction (direct or not) between ORF23 and ORF141 was further confirmed by co-IP assay. The enzyme activity analysis showed that activities of RR were only detected when the reaction solutions contained both ORF23 and ORF141 proteins of CyHV-2, which confirmed the hypothesis that ORF23 protein, together with ORF141 protein function as RR in CyHV-2.

With siRNA interference of the expression of ORF23 and/or ORF141 genes, the copy number and capsid protein ORF72 of CyHV-2 were significantly reduced in virus-infected cells. A previous study reported that siRNA interference with the expression of the RR large subunit (UL39) effectively inhibited the replication of HSV-1 (Ren et al., 2008). One of the more important findings of the in-depth study showed that siRNA inhibited HSV-1 replication by up to 73% *via* interfering with the expression of RR (Ren et al., 2011). Although siRNA significantly decreased the mRNA expression of ORF23 and ORF141 genes in this study, the inhibition of CyHV-2 replication is limited, which may be due to the difference of cell cycle. It was reported that replication of the ICP6 mutant of HSV-1 was unaffected in dividing cells, but inhibited in non-dividing cells (Goldstein and Weller, 1988). Moreover, some herpesviruses have evolved strategies to induce changes in cell cycle to increase the levels of dNTP in infected cells. It has conclusively been shown that the major immediate-early proteins of human cytomegalovirus (HCMV) were able to induce cell cycle progression from the G0/G1 phase to the S phase by targeting RB protein (Castillo and Kowalik, 2002). Another interesting discovery was that herpesvirus induced the expression of the cellular RR in HELF cells. At 3 hpi, the protein levels of cellular RR were detectable and the expression trends of both subunits were consistent in HCMV-infected HELF cells (Patrone et al., 2003). Previous studies on Herpesviridae RR found that deletion of ORF141 gene delayed and slowed CyHV-3 replication, but did not prevent the virus titer from reaching the control level (Fuchs et al., 2011). According to this study, CyHV-2 RR encoded by ORF23 and ORF141 genes was verified and the progress replication of CyHV-2 was suppressed *via* interfering with the expression of these two genes. Although herpesviruses have various mechanisms to regulate the dNTP levels in infected cells, and only inhibiting the RR encoded by CyHV-2 cannot significantly suppress viral replication, CyHV-2 ORF23 and ORF141 can still serve as a therapeutic target for inhibiting the virus.

Hydroxyurea, a free radical scavenger, acts as an inhibitor of RR. It prevents the catalytic action of the RR holocomplex *via* quenching the tyrosyl radical of RR2 (Lassmann et al., 1992). The current study found that hydroxyurea was able to reduce intracellular dNTP levels by inhibiting activities of both viral and host RRs, which eventually suppressed DNA synthesis and repair. Hydroxyurea has been widely investigated in the treatment of human viral disease. *In vitro* studies have shown that hydroxyurea alone or in combination with anti-HCV drugs (such as IFN-α) was effective against HCV. The antiviral effect of hydroxyurea was not due to the cytotoxic effects, but the inhibition of the activities of RRs which resulted in the reduction of dNTP levels in host cells (Nozaki et al., 2010). *In vitro* and *in vivo* studies have shown that hydroxyurea inhibited HBV replication by reducing intracellular dNTP levels (Cohen et al., 2010). Hydroxyurea also inhibited herpes simplex virus (HSV) which is resistant to nucleoside analog Aciclovir and Cidofovir. *In vitro* studies have shown that hydroxyurea, together with Aciclovir and Cidofovir, had synergistic antiviral effects on HSV mutants with mutations in genes encoding thymidine kinase and/or DNA polymerase (Sergerie and Boivin, 2008).

In this study, only the addition of high concentrations of hydroxyurea inhibited the replication process of the CyHV-2 than the lower concentrations and caused a significant toxic effect on the cellular activity of GICF. It has been found that hydroxyurea may be toxic to cells because of its reduced stability in water. Over time or upon heating, hydroxyurea decomposes to N-hydroxyurethane, hydrogen cyanide, nitric oxide, and/or hydrogen peroxide (Hallmark et al., 2021). Hydroxyurea may also indirectly induce the generation of other reactive oxygen species (ROS) through interactions with iron or other metals (Huang et al., 2016). In a previous study (Lu et al., 2022), that intracellular levels of ROS were found increased during replication of CyHV-2, while antioxidants could inhibit viral replication by inhibiting the rise of ROS, which may support the reason why low concentrations of hydroxyurea does not suppress CyHV-2 replication and high concentrations of hydroxyurea only partially inhibits CyHV-2 replication.

In conclusion, this study indicated that the CyHV-2 proteins ORF23 and ORF141 function as viral RR and their function makes an effect to CyHV-2 replication. Although inhibition of CyHV-2-encoded RR with hydroxyurea is only limited, the proteins encoded by ORF23 and ORF141 can still serve as targets for inhibition of CyHV-2 replication. With further research, the molecular targets for inhibition of ORF23 and ORF141 interaction will be investigated to antagonize viral replication in CyHV-2 infected hosts.

## Data availability statement

The datasets presented in this study can be found in online repositories. The names of the repository/repositories and accession number(s) can be found in the article/Supplementary material.

## Author contributions

DX: conceptualization and methodology. WC, QC, and DX: investigation. YR: visualization. LL and DX: funding acquisition. YZ, LL, LG, and DX: project administration and supervision. WC, QC, YZ, and DX: writing—original draft. WC, YZ, and DX: writing—review and editing. All authors read and approved the final version of the manuscript.
