# Supplementary material for: The identification of viral ribonucleotide reductase encoded by ORF23 and ORF141 genes and effect on CyHV-2 replication
Source: Front Microbiol. 2023 Apr 18;14:1154840. doi: 10.3389/fmicb.2023.1154840 (PMC10151572; doi:10.3389/fmicb.2023.1154840)
Supplement: Supplementary file 1 [file Presentation_1.pdf]

Supplementary Material

The identification of viral Ribonucleotide reductase encoded by *ORF23* and *ORF141* genes and effect on CyHV-2 replication

Wenjie Cheng[1,2,3,#], Qikang Chen[1,2,3,#], Yilin Ren[1,2,3], Ye Zhang[1,2,3,4], Liqun Lu[1,2,3,4], Lang Gui[1,2,3,4] and Dan Xu[1,2,3,4*]

*** Correspondence:** dxu@shou.edu.cn (D. Xu).

1 Supplementary Figures and Tables

1.1 Supplementary Figures

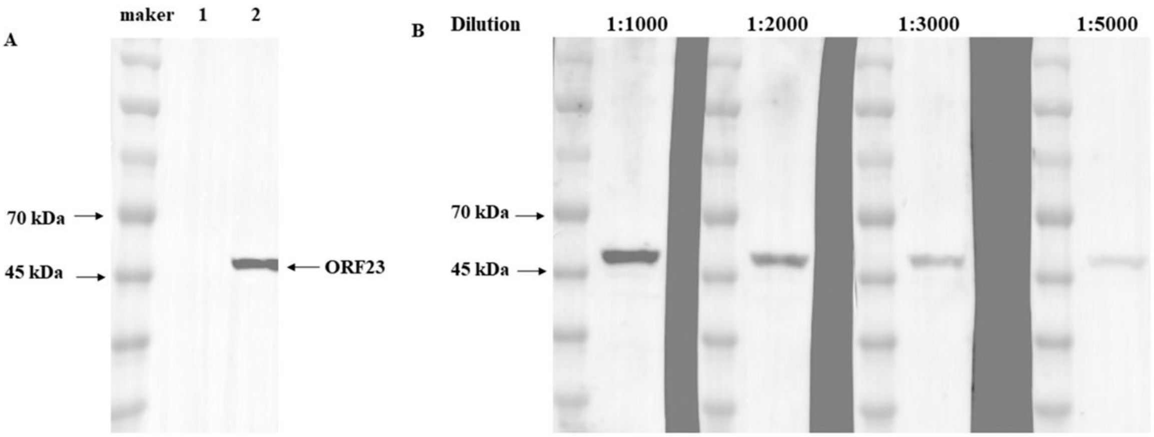

FIGURE S1. Validation of the ORF23 pAb by Western blot. (A) Lanes "M", "1" and "2" represent protein maker, total protein of GiCF cells, and total protein of GiCF cells infected with CyHV-2, respectively. （B） The antibodies recognized ORF23 encoded proteins at dilutions of 1: 1000,1:2000,1:3000 and 1:5000, respectively.

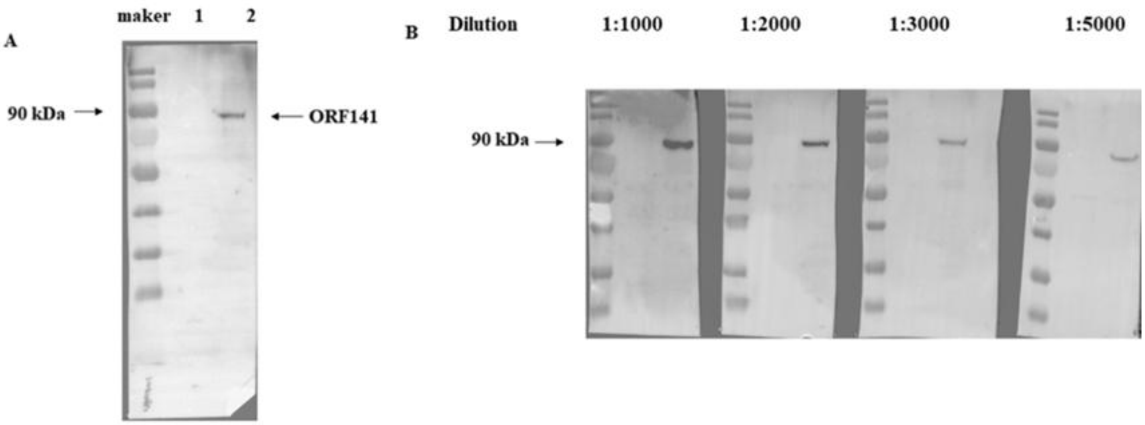

FIGURE S2. Validation of the ORF141 pAb by Western blot. (A) Lanes "M", "1" and "2" represent protein maker, total protein of GiCF cells, and total protein of GiCF cells infected with CyHV-2, respectively. （B） The antibodies recognized ORF141 encoded proteins at dilutions of 1: 1000,1:2000,1:3000 and 1:5000, respectively.

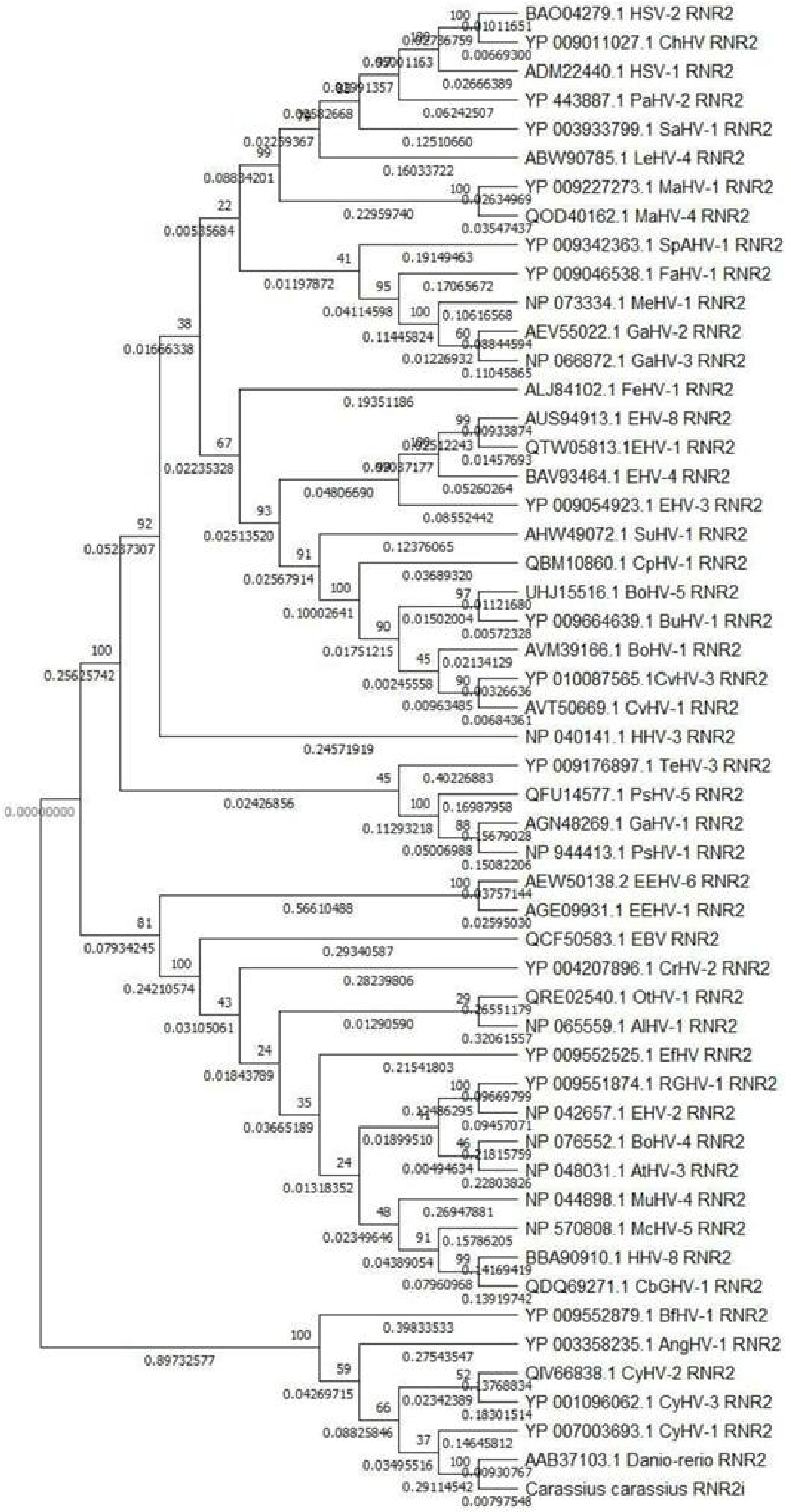

FIGURE S3. Results of statistical analysis related to RNR2 phylogenetic tree.

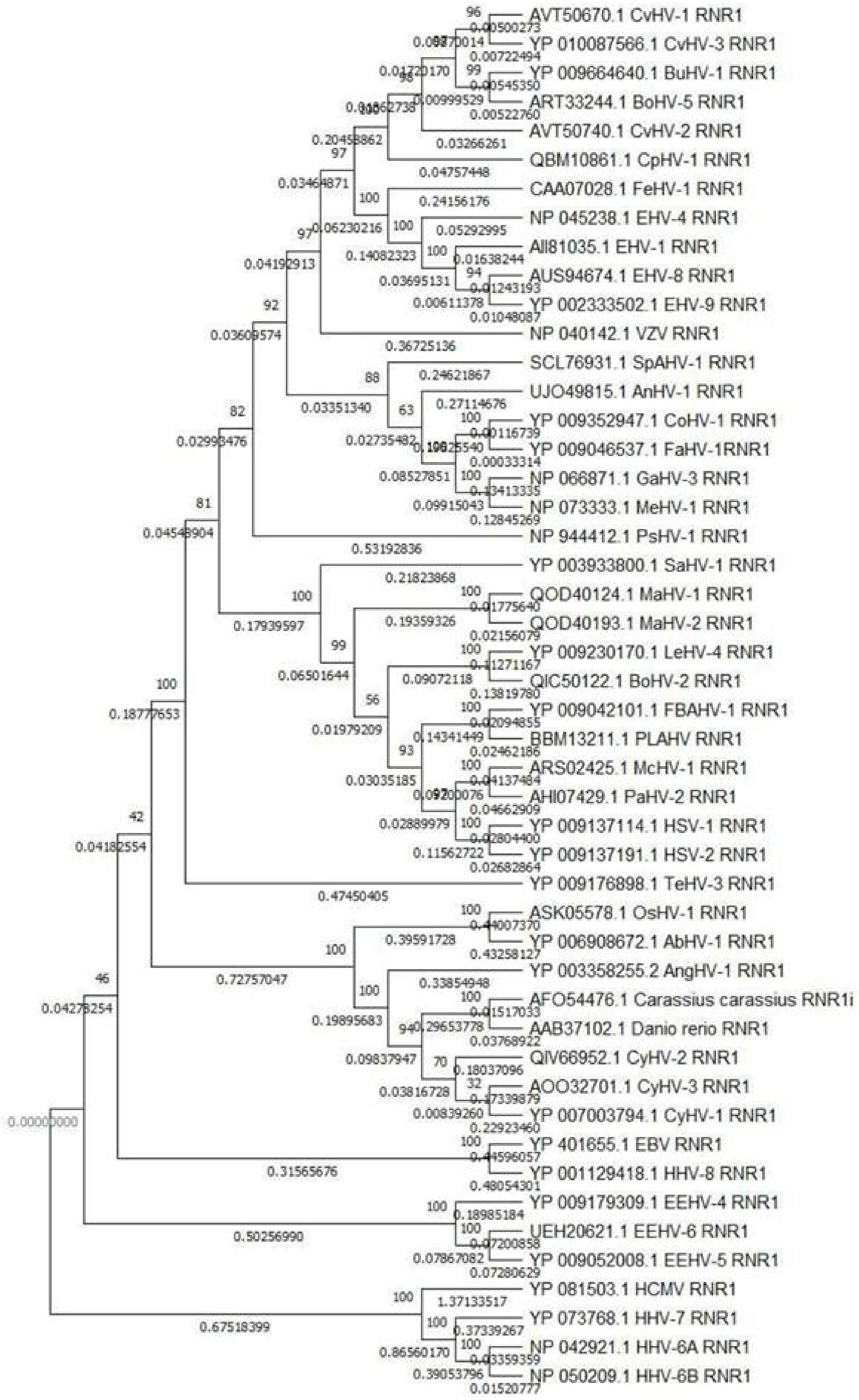

FIGURE S4. Results of statistical analysis related to RNR1 phylogenetic tree.

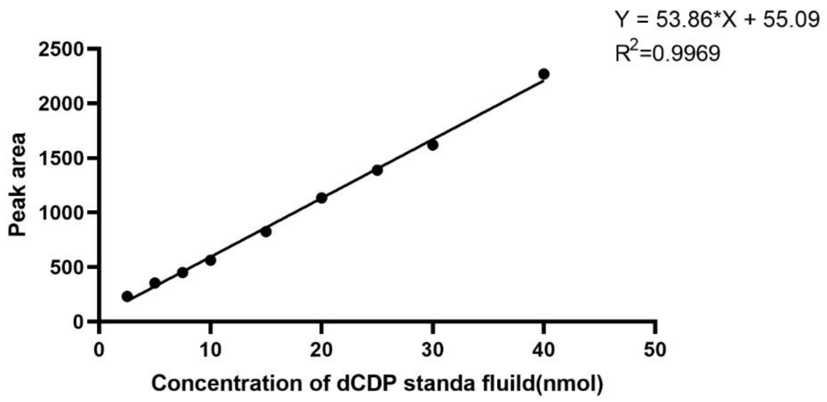

FIGURE S5. Standard curve of dCDP and Ribonucleotide reductase activity assay. Standard curves constructed using different concentrations of dCDP standards.

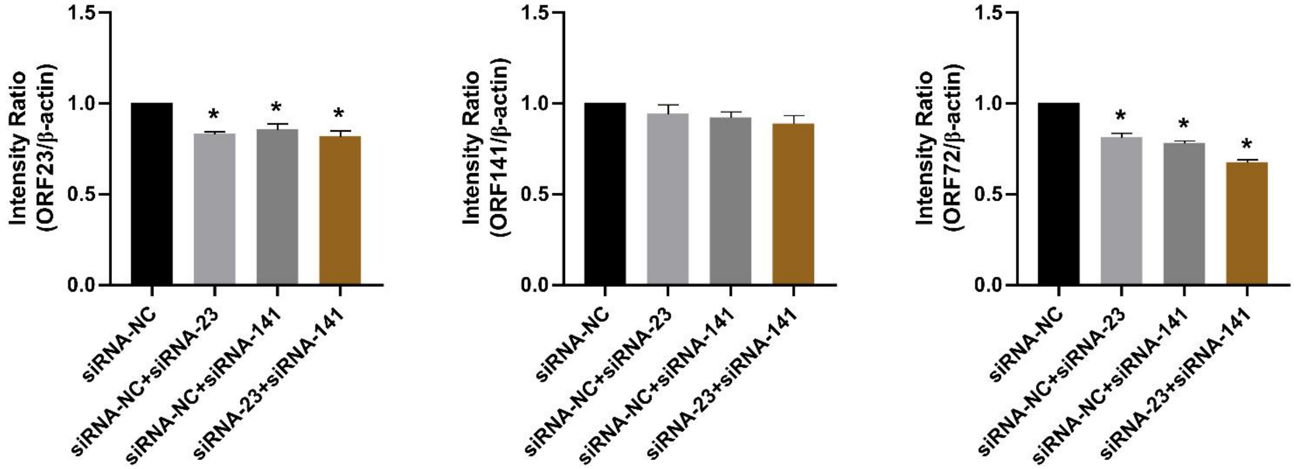

FIGURE S6. The results of protein normalization with β-actin as an internal reference protein in Figure 7C,*$P < 0.05$ vs. NC group.

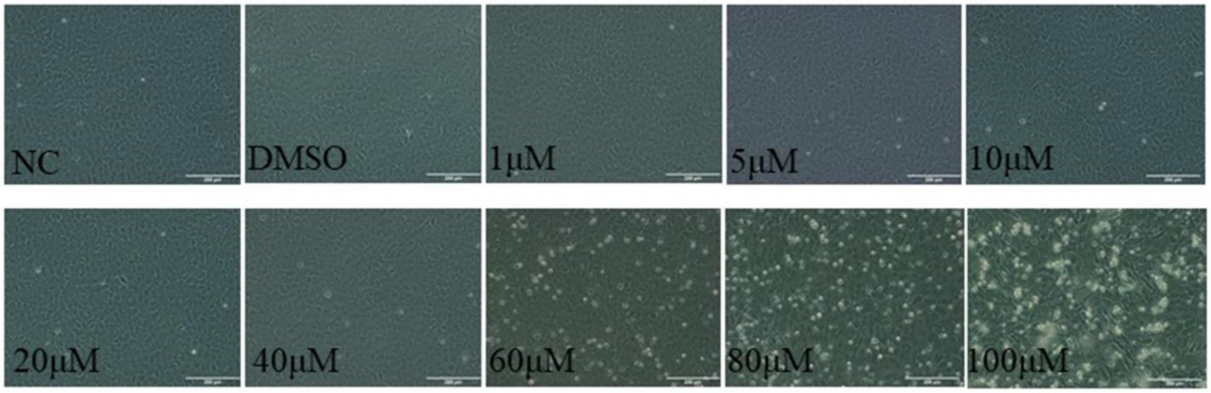

FIGURE S7. The effects of different dosages of hydroxyurea (0, 1, 5, 10, 20, 40, 60, 80, and 100μM) on GICF cells were observed by microscope.

1.2 Supplementary Tables

TABLE S1. polymerase chain reaction and real-time fluorescent quantitative primers.

Gene	Primer sequence(5’ to 3’)
pGEX-23-F	tggatccccgaattcATGGAAGTCGATATGCAAGACC
pGEX-23-R	atgcggccgctcgagAAATTCAAATGAATCGTCCTCT
pET-141F	ggtcgcggatccgaattcATGGAATCAATCACCACTACTAC CGTTGGC
pET-141-R	tggtggtggtggtgctcgagGCTAGAACACATGACACACAC ATCATC
pGEX-4T-3-F	GCGGCCGCATCGTGACTG
pGEX-4T-3-R	GAATTCGGGGATCCACGC
pET-28a-F	CTCGAGCACCACCACCACC
pET-28a-R	GAATTCGGATCCGCGACC
pDsRed-23-F	gtcagatccgctagcATGGAAGTCGATATGCAAGACC
pDsRed-23-R	tcgactgcagaattcAAATTCAAATGAATCGTCCTCT
pEGFP-141-F	gtcagatccgctagcATGGAATCAATCACCACTACTA
pEGFP-141-R	tcgactgcagaattcGCTAGAACACATGACACACACA

pCMV-23-F	gccatggaggaattcATGGAAGTCGATATGCAAGACC
pCMV-23-R	gatccccgcggccgcAAATTCAAATGAATCGTCCTCT
pcDNA-141-F	tcgaatagactcgagATGGAATCAATCACCACTACTA
PcDNA-141-R	agtgtggtggaattcGCTAGAACACATGACACACACA
pDsRed-N1-F	GAATTCTGCAGTCGACGGTACC
pDsRed-N1-R	GCTAGCGGATCTGACGGTTCA
pEGFP-N1-F	GAATTCTGCAGTCGACGGTACC
pEGFP-N1-R	GCTAGCGGATCTGACGGTTCA
pCMV-HA-F	GCGGCCGCGGGGATCCAGAC
pCMV-HA-R	GAATTCCTCCATGGCCATAA
pcDNA3.1-3xflag-F	GAATTCCACCACACTGGACT
pcDNA3.1-3xflag-R	CTCGAGTCTAttcGAGGGCCC
ORF23-q-F	GGACTACGAGCCTTCTGCTG
ORF23-q-R	GATAGAGCGTACCAGCCGTC
ORF141-q-F	TGGATCCCGGACCTCTTCAT
ORF141-q-R	GTCCTCTTGACTGCACCCTC
ORF72-q-F	GCGGATACGTTGGACGATCT
ORF72-q-R	CTCGGCTCTGATGGTGTTGT
β-actin-F	CACTGTGCCCATCTACGAG

β-actin-R	CCATCTCCTGCTCGAAGTC

TABLE S2. siRNA sequence.

siRNA	Sense(5'-3')	Antisense(5'-3')
siRNA-23	GCAGGUAACACACAAAGAUTT	AUCUUUGUGUGUUACCUGCTT
141-SiRNA-999	GGACCAGAGAUCUCUUUCATT	UGAAAGAGAUCUCUGGUCCTT
141-SiRNA-1236	GUCCCUUUAUGCUCUACAATT	UUGUAGAGCAUAAAGGGACTT
SiRNA-NC	UUCUCCGAACGUGUCACGUT	UUCUCCGAACGUGUCACGUTT
